# Discrete water clusters in tetra-μ-cyanido-tetra­cyanidobis(1,4,7-triisopropyl-1,4,7-triaza­cyclo­nona­ne)dicopper(II)dinickel(II) tetra­hydrate

**DOI:** 10.1107/S1600536812024282

**Published:** 2012-06-13

**Authors:** Hong-Xia Cui, Yan-Chao Wang

**Affiliations:** a Department of Chemistry and Chemical Engineering, Shengli College, China University of Petroleum, Dongying 257097, People’s Republic of China; b Department of Basic Science, Liao Ning Institute of Science and Technology, Benxi 117004, People’s Republic of China

## Abstract

The title tetra­cyanido­nickelate–copper complex, [Cu_2_Ni_2_(CN)_8_(C_15_H_33_N_3_)_2_]·4H_2_O, was synthesized by self-assembly using potassium tetracyanidonickelate(II) and dichlorido(1,4,7-triisopropyl-1,4,7-triazacyclononane)copper(II). The asymmetric unit contains half of a complex mol­ecule and two water mol­ecules. The entire complex has -1 symmetry and contains Ni(II) in a slightly distorted square-planar and Cu(II) in a square-pyramidal coordination environment. The crystal packing shows a discrete tetra­mer water cluster. Within the cluster, the four water mol­ecules are fully coplanar and each water monomer acts both as single O—H⋯O and O—H⋯N hydrogen-bond donor and acceptor.

## Related literature

For properties and applications of cyanide-bridged coordination complexes, see: Zhao *et al.* (2009 ▶); Dunbar & Heintz (1997 ▶); Orendac *et al.* (2002 ▶). For the use of the tetra­cyanido­nickelate anion as a bridging ligand in the construction of one-, two- and three-dimensional structures, see: Bozoglian *et al.* (2005 ▶); Maji *et al.* (2001 ▶); Dunbar & Heintz (1997 ▶); Černák *et al.* (1988 ▶, 1990 ▶); Černák & Abboud (2000 ▶). For the influence on water aggregations of the overall structure of their surroundings, see: Long *et al.* (2004 ▶); Xantheas (1995 ▶). For water clusters, see: Ugalde *et al.* (2000 ▶); Gregory & Clary (1996 ▶). For the synthesis of the ligand, see: Hay & Norman (1979 ▶). Chen *et al.* (2009 ▶).
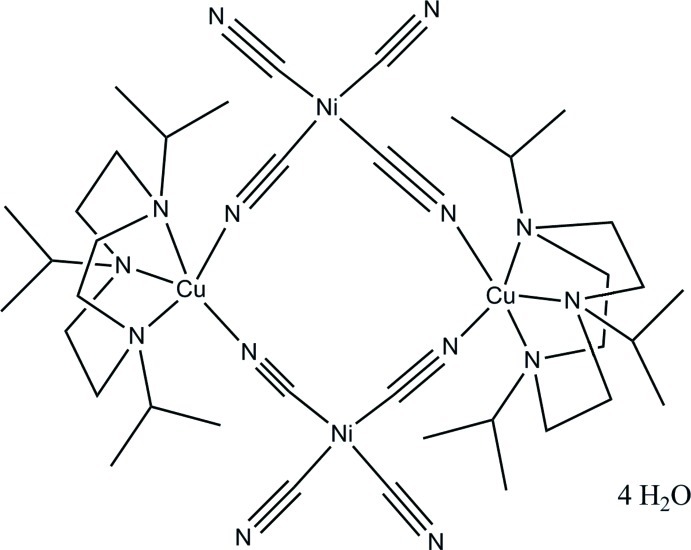


## Experimental

### 

#### Crystal data

[Cu_2_Ni_2_(CN)_8_(C_15_H_33_N_3_)_2_]·4H_2_O
*M* *_r_* = 1035.59
Monoclinic, 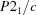

*a* = 8.5896 (17) Å
*b* = 18.092 (4) Å
*c* = 15.615 (3) Å
β = 95.61 (3)°
*V* = 2415.1 (8) Å^3^
*Z* = 2
Mo *K*α radiation
μ = 1.69 mm^−1^
*T* = 293 K
0.14 × 0.12 × 0.06 mm

#### Data collection

Bruker P4 diffractometer
Absorption correction: multi-scan (*XSCANS*; Bruker, 1999 ▶) *T*_min_ = 0.798, *T*_max_ = 0.906
18582 measured reflections
5622 independent reflections
4475 reflections with *I* > 2σ(*I*)
*R*_int_ = 0.039
3 standard reflections every 120 min intensity decay: 1.0%

#### Refinement

*R*[*F* ^2^ > 2σ(*F* ^2^)] = 0.034
*wR*(*F* ^2^) = 0.074
*S* = 1.03
5622 reflections
293 parameters
6 restraints
H atoms treated by a mixture of independent and constrained refinement
Δρ_max_ = 0.52 e Å^−3^
Δρ_min_ = −0.39 e Å^−3^

### 

Data collection: *XSCANS* (Bruker, 1999 ▶); cell refinement: *XSCANS*; data reduction: *XSCANS*; program(s) used to solve structure: *SHELXS97* (Sheldrick, 2008 ▶); program(s) used to refine structure: *SHELXL97* (Sheldrick, 2008 ▶); molecular graphics: *SHELXTL* (Sheldrick, 2008 ▶); software used to prepare material for publication: *SHELXTL*.

## Supplementary Material

Crystal structure: contains datablock(s) I, global. DOI: 10.1107/S1600536812024282/vm2171sup1.cif

Structure factors: contains datablock(s) I. DOI: 10.1107/S1600536812024282/vm2171Isup2.hkl

Additional supplementary materials:  crystallographic information; 3D view; checkCIF report

## Figures and Tables

**Table 1:** Hydrogen-bond geometry (Å, °)

*D*—H⋯*A*	*D*—H	H⋯*A*	*D*⋯*A*	*D*—H⋯*A*
O1—H1*A*⋯N3^i^	0.85 (2)	2.02 (2)	2.874 (3)	179 (3)
O1—H1*B*⋯O2^ii^	0.85 (2)	1.92 (2)	2.745 (3)	165 (2)
O2—H2*A*⋯N2	0.86 (2)	1.98 (2)	2.831 (3)	171 (3)
O2—H2*B*⋯O1^iii^	0.86 (3)	1.92 (3)	2.775 (3)	174 (2)

Symmetry codes: (i) 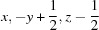; (ii) 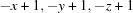; (iii) 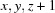.

**Fig. 1.**
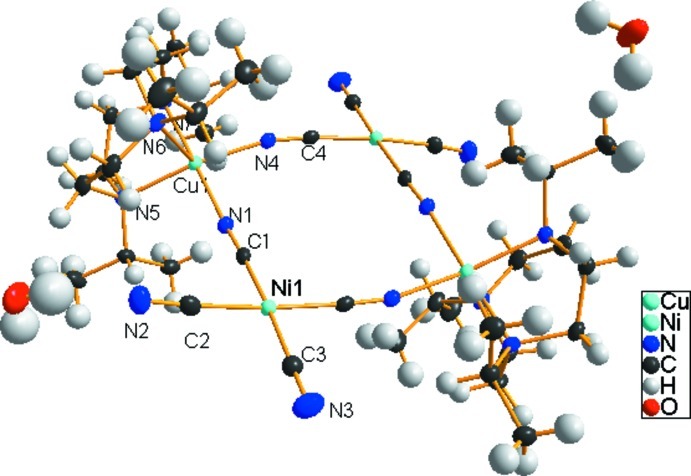
The molecular structure of 1 showing 30% probability displacement ellipsoids for non-H atoms. The second half of the molecule is generated by symmetry code -x, -y - 1, -z - 1.

**Fig. 2.**
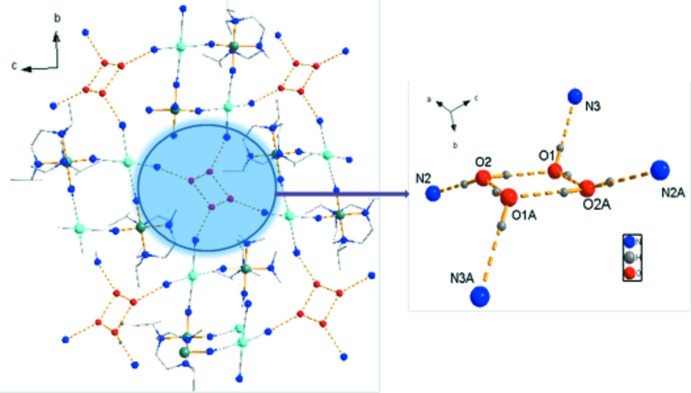
Stacking diagram of 1 and hydrogen bonding in the water cluster (symmetry code A: 1 - x, 1 - y, -z).

## supplementary crystallographic information

### Comment

In recent years, much attention has been paid to assemble cyanide-bridged coordination complexes because of their promising properties and applications including electronics, magnetism and catalysis (Zhao *et al.*, 2009; Dunbar & Heintz, 1997; Orendac *et al.*, 2002), in which tetracyanonickelate complexes have also become the focus. On the one hand, diamagnetic [Ni(CN)_4_]^2-^ is an excellent model for magnetic studies which bridge paramagnetic ions, but on the other hand the tetracyanonickelate anion, as a bridging ligand, can be used to construct one-dimensional, two-dimensional and three-dimensional structures (Bozoglian *et al.*, 2005; Maji *et al.*, 2001; Dunbar & Heintz, 1997; Černák *et al.*, 2000; 1988; 1990). Low-dimensional cyanide-bridged complexes based on [Ni(CN)_4_]^2-^ form a new family of molecular magnetic materials. However, the use of macrocyclic ligands as terminal group to control the low-dimensional structure is still relatively rare. On the other hand, water clusters can play an important role in the stabilization of supramolecular systems both in solution and in the solid state, and there is clearly a need for a better understanding of how such water aggregations are influenced by the overall structure of their surroundings (Long *et al.*, 2004; Xantheas, 1995). In the past several decades, considerable attention has been focused on theoretical and experimental studies of small water clusters to understand the structures and characteristics of liquid water and ice (Ugalde *et al.*, 2000; Gregory *et al.*, 1996).

In this study, we report a complex 1 in which [Ni(CN)_4_]^2-^ acts as bridging ligand to construct a low-dimensional complex. Complex 1 can be synthesized by the reaction of [Ni(Pr_3_TACN)]Cl_2_ with K_2_[Ni(CN)_4_], which is a cyanide bridged [2 + 2] type of molecular square. The ligand 1,4,7-triisopropyl-1,4,7-triazacyclononane (Pr_3_TACN) was synthesized according to the literature (Hay *et al.*, 1979; Chen *et al.*, 2009). The structure of the complex 1 is shown in Figure 1. The complex contains two [Ni(CN)_4_]^2-^ bridges and two *cis*-[Cu(Pr_3_TACN)]^2+^ moieties in *cis*-positions to form a [2 + 2] type of discrete molecular square. The Cu1—N(macrocycle) distances (2.0686 (17)–2.2153 (18) Å) are close to the Cu1-N(cyano) distances (1.9781 (18) and 1.9929 (17) Å) and they are longer than the Ni1—C(cyano) distances (1.861 (2)–1.871 (2) Å). Furthermore the C—N(coordinated) distances of the cyano groups are close to the C—N(uncoordinated) distances. Interestingly, a cyclic water tetramer is located in between the complexes 1. Within the cluster, the four water molecules are fully coplanar and each water monomer acts as both single hydrogen bond donor and acceptor. The hydrogen bond distances and angles within the water tetramer are as follows: O1—O2^i^ = 2.775 (3) Å, O1—O2^ii^ = 2.745 (3) Å, O1^i^—O2—O1^iii^ = 100.05 (9)°, O2^i^—O1—O2^ii^ = 79.95 (8)° (symmetry codes: (i) *x*, *y*, -1 + *z*; (2) 1 - *x*, 1 -*y*, 1 -*z*; (iii) *x*,*y*, *z* + 1). The average hydrogen bond distance within the water tetramer is 2.76 (1) Å, which is slightily shorter than 2.78 Å estimated in the udud water tetramer of (D_2_O)_4_ (Ugalde *et al.*, 2000). The most remarkable feature in 1 is that the cyclic water tetramer connects the [2 + 2] molecular square through hydrogen bonds to form a two-dimensional structure (Fig. 2, Table 1).

### Experimental

A water solution (25 ml) of potassium tetracyanonickel (0.111 g, 0.4 mmol) was layered with an acetonitrile solution (25 ml) of dichloro-(1,4,7-triisopropyl-1,4,7-triazcyclononane)-copper(II) (0.151 g, 0.4 mmol). After about 3 weeks, prism-shaped blue crystals of 1 formed from the solution. The crystals were collected, washed with water and methanol, and dried in the air. Yield: 45% (based on tetracyanonickelate salts). Anal. Calcd for C_19_H_37_N_7_CuNiO_2_: C, 44.07; H, 7.20; N, 18.93. Found: C, 44.25; H, 7.25; N, 19.02%. IR (KBr, cm^-1^): 3455 (s), 2974 (s), 2164 (CN, coordinated) and 2135 (CN, uncoordinated), 1652 (s).

### Refinement

A total of 6 similarity restraints were used for the H atoms of the water molecules which were initially refined with fixed O—H distances of 0.85 Å and 1.2*U*_eq_(O). The other H atoms were placed in calculated positions and refined as riding on the parent C atoms with C—H = 0.93–0.97 Å and *U*_iso_(H) = 1.2 *U*_eq_ (C).

### Crystal data

**Table:**

[Cu_2_Ni_2_(CN)_8_(C_15_H_33_N_3_)_2_]·4H_2_O	*F*(000) = 1092
*M**_r_* = 1035.59	*D*_x_ = 1.424 Mg m^−^^3^
Monoclinic, *P*2_1_/*c*	Mo *K*α radiation, λ = 0.71073 Å
Hall symbol: -P 2ybc	Cell parameters from 3989 reflections
*a* = 8.5896 (17) Å	θ = 2.0–25.5°
*b* = 18.092 (4) Å	µ = 1.69 mm^−^^1^
*c* = 15.615 (3) Å	*T* = 293 K
β = 95.61 (3)°	Prism, blue
*V* = 2415.1 (8) Å^3^	0.14 × 0.12 × 0.06 mm
*Z* = 2	

### Data collection

**Table:**

Bruker P4 diffractometer	4475 reflections with *I* > 2σ(*I*)
Radiation source: fine-focus sealed tube	*R*_int_ = 0.039
Graphite monochromator	θ_max_ = 27.9°, θ_min_ = 1.7°
ω scans	*h* = −11→10
Absorption correction: multi-scan (*XSCANS*; Bruker, 1999)	*k* = −23→23
*T*_min_ = 0.798, *T*_max_ = 0.906	*l* = −20→12
18582 measured reflections	3 standard reflections every 120 min
5622 independent reflections	intensity decay: 1.0%

### Refinement

**Table:**

Refinement on *F*^2^	Primary atom site location: structure-invariant direct methods
Least-squares matrix: full	Secondary atom site location: difference Fourier map
*R*[*F*^2^ > 2σ(*F*^2^)] = 0.034	Hydrogen site location: inferred from neighbouring sites
*wR*(*F*^2^) = 0.074	H atoms treated by a mixture of independent and constrained refinement
*S* = 1.03	*w* = 1/[σ^2^(*F*_o_^2^) + (0.0357*P*)^2^] where *P* = (*F*_o_^2^ + 2*F*_c_^2^)/3
5622 reflections	(Δ/σ)_max_ = 0.002
293 parameters	Δρ_max_ = 0.52 e Å^−^^3^
6 restraints	Δρ_min_ = −0.39 e Å^−^^3^

### Special details

**Table:**

Geometry. All e.s.d.'s (except the e.s.d. in the dihedral angle between two l.s. planes) are estimated using the full covariance matrix. The cell e.s.d.'s are taken into account individually in the estimation of e.s.d.'s in distances, angles and torsion angles; correlations between e.s.d.'s in cell parameters are only used when they are defined by crystal symmetry. An approximate (isotropic) treatment of cell e.s.d.'s is used for estimating e.s.d.'s involving l.s. planes.
Refinement. Refinement of *F*^2^ against ALL reflections. The weighted *R*-factor *wR* and goodness of fit *S* are based on *F*^2^, conventional *R*-factors *R* are based on *F*, with *F* set to zero for negative *F*^2^. The threshold expression of *F*^2^ > σ(*F*^2^) is used only for calculating *R*-factors(gt) *etc*. and is not relevant to the choice of reflections for refinement. *R*-factors based on *F*^2^ are statistically about twice as large as those based on *F*, and *R*- factors based on ALL data will be even larger.

### Fractional atomic coordinates and isotropic or equivalent isotropic displacement parameters (Å^2^)

**Table:**

	*x*	*y*	*z*	*U*_iso_*/*U*_eq_	
Cu1	0.14378 (3)	0.639359 (12)	0.659167 (15)	0.01450 (7)	
Ni1	0.20048 (3)	0.370161 (13)	0.612942 (16)	0.01552 (8)	
N1	0.12720 (19)	0.52998 (9)	0.64625 (11)	0.0183 (4)	
N2	0.4785 (2)	0.39240 (11)	0.74309 (12)	0.0336 (5)	
N3	0.2998 (2)	0.21257 (10)	0.58992 (14)	0.0358 (5)	
N4	−0.0304 (2)	0.35220 (9)	0.45692 (11)	0.0188 (4)	
N5	0.18877 (19)	0.63639 (9)	0.79170 (10)	0.0164 (4)	
N6	0.40058 (19)	0.63663 (9)	0.65419 (11)	0.0200 (4)	
N7	0.16954 (19)	0.75278 (9)	0.67369 (10)	0.0173 (4)	
C1	0.1467 (2)	0.46860 (11)	0.63196 (13)	0.0173 (4)	
C2	0.3701 (3)	0.38215 (11)	0.69594 (13)	0.0220 (5)	
C3	0.2615 (2)	0.27259 (12)	0.59875 (14)	0.0225 (5)	
C4	0.0494 (2)	0.35903 (10)	0.51975 (13)	0.0174 (4)	
C5	0.3626 (2)	0.62659 (12)	0.81156 (14)	0.0226 (5)	
H5A	0.3815	0.5925	0.8594	0.027*	
H5B	0.4083	0.6738	0.8296	0.027*	
C6	0.4447 (2)	0.59780 (12)	0.73657 (13)	0.0239 (5)	
H6A	0.5568	0.6024	0.7507	0.029*	
H6B	0.4209	0.5457	0.7289	0.029*	
C7	0.4476 (2)	0.71567 (12)	0.65622 (15)	0.0252 (5)	
H7A	0.5301	0.7228	0.6188	0.030*	
H7B	0.4891	0.7287	0.7143	0.030*	
C8	0.3109 (3)	0.76665 (11)	0.62750 (14)	0.0234 (5)	
H8A	0.3438	0.8175	0.6370	0.028*	
H8B	0.2832	0.7602	0.5662	0.028*	
C9	0.2005 (3)	0.77059 (11)	0.76740 (13)	0.0206 (5)	
H9A	0.1495	0.8168	0.7793	0.025*	
H9B	0.3121	0.7769	0.7820	0.025*	
C10	0.1412 (3)	0.71016 (11)	0.82235 (13)	0.0205 (5)	
H10A	0.1833	0.7171	0.8817	0.025*	
H10B	0.0281	0.7127	0.8198	0.025*	
C11	0.4573 (2)	0.59355 (13)	0.58066 (14)	0.0256 (5)	
H11	0.4070	0.5449	0.5806	0.031*	
C12	0.4061 (3)	0.62923 (13)	0.49380 (15)	0.0331 (6)	
H12A	0.2950	0.6372	0.4888	0.050*	
H12B	0.4322	0.5972	0.4484	0.050*	
H12C	0.4587	0.6757	0.4897	0.050*	
C13	0.6336 (3)	0.58003 (14)	0.59072 (17)	0.0376 (6)	
H13A	0.6875	0.6265	0.5960	0.056*	
H13B	0.6632	0.5541	0.5412	0.056*	
H13C	0.6610	0.5509	0.6414	0.056*	
C14	0.0316 (2)	0.79650 (11)	0.63208 (14)	0.0219 (5)	
H14	0.0216	0.7844	0.5706	0.026*	
C15	0.0510 (3)	0.88029 (11)	0.63950 (15)	0.0279 (5)	
H15A	0.0495	0.8949	0.6985	0.042*	
H15B	−0.0331	0.9042	0.6051	0.042*	
H15C	0.1489	0.8945	0.6195	0.042*	
C16	−0.1201 (3)	0.77345 (12)	0.66699 (14)	0.0273 (5)	
H16A	−0.1295	0.7206	0.6650	0.041*	
H16B	−0.2070	0.7953	0.6326	0.041*	
H16C	−0.1199	0.7900	0.7254	0.041*	
C17	0.1007 (3)	0.57566 (11)	0.83338 (13)	0.0215 (5)	
H17	0.1460	0.5283	0.8183	0.026*	
C18	−0.0718 (3)	0.57506 (12)	0.79870 (14)	0.0253 (5)	
H18A	−0.1217	0.6189	0.8173	0.038*	
H18B	−0.1217	0.5323	0.8201	0.038*	
H18C	−0.0804	0.5736	0.7370	0.038*	
C19	0.1143 (3)	0.58101 (12)	0.93095 (13)	0.0300 (6)	
H19A	0.2220	0.5877	0.9523	0.045*	
H19B	0.0754	0.5364	0.9544	0.045*	
H19C	0.0540	0.6223	0.9478	0.045*	
O1	0.3097 (2)	0.42588 (10)	0.00040 (11)	0.0420 (5)	
O2	0.5596 (2)	0.45720 (11)	0.90642 (13)	0.0454 (5)	
H1A	0.308 (3)	0.3849 (7)	0.0270 (14)	0.058 (10)*	
H1B	0.340 (3)	0.4592 (9)	0.0362 (12)	0.048 (9)*	
H2A	0.542 (3)	0.4407 (18)	0.8550 (9)	0.095 (14)*	
H2B	0.481 (3)	0.4447 (17)	0.9337 (16)	0.075 (12)*	

### Atomic displacement parameters (Å^2^)

**Table:**

	*U*^11^	*U*^22^	*U*^33^	*U*^12^	*U*^13^	*U*^23^
Cu1	0.01485 (13)	0.01184 (12)	0.01636 (14)	−0.00097 (10)	−0.00065 (9)	−0.00208 (10)
Ni1	0.01783 (14)	0.01198 (13)	0.01623 (15)	0.00133 (10)	−0.00106 (10)	−0.00066 (10)
N1	0.0188 (9)	0.0156 (9)	0.0200 (9)	−0.0002 (7)	−0.0007 (7)	−0.0017 (7)
N2	0.0328 (12)	0.0317 (11)	0.0337 (12)	0.0063 (9)	−0.0098 (9)	−0.0029 (9)
N3	0.0373 (12)	0.0190 (10)	0.0513 (14)	0.0058 (9)	0.0058 (10)	−0.0024 (9)
N4	0.0203 (9)	0.0149 (9)	0.0209 (10)	−0.0008 (7)	0.0010 (7)	−0.0016 (7)
N5	0.0179 (9)	0.0143 (8)	0.0168 (9)	0.0009 (7)	−0.0001 (7)	−0.0013 (7)
N6	0.0158 (9)	0.0192 (9)	0.0250 (10)	−0.0034 (7)	0.0026 (7)	−0.0059 (7)
N7	0.0194 (9)	0.0133 (8)	0.0194 (9)	−0.0004 (7)	0.0025 (7)	0.0000 (7)
C1	0.0146 (10)	0.0211 (11)	0.0155 (10)	−0.0035 (8)	−0.0020 (8)	−0.0003 (8)
C2	0.0271 (12)	0.0169 (11)	0.0218 (12)	0.0051 (9)	0.0018 (9)	0.0006 (9)
C3	0.0234 (12)	0.0212 (11)	0.0227 (12)	0.0000 (9)	0.0013 (9)	0.0005 (9)
C4	0.0196 (11)	0.0107 (10)	0.0222 (12)	0.0000 (8)	0.0041 (8)	−0.0009 (8)
C5	0.0171 (11)	0.0240 (12)	0.0250 (12)	0.0010 (9)	−0.0067 (9)	−0.0009 (9)
C6	0.0149 (11)	0.0241 (12)	0.0315 (13)	0.0002 (9)	−0.0030 (9)	−0.0051 (10)
C7	0.0210 (12)	0.0236 (12)	0.0317 (13)	−0.0074 (9)	0.0057 (10)	−0.0072 (10)
C8	0.0274 (12)	0.0198 (11)	0.0241 (12)	−0.0080 (9)	0.0083 (9)	−0.0018 (9)
C9	0.0242 (11)	0.0161 (10)	0.0209 (11)	−0.0013 (9)	−0.0004 (9)	−0.0056 (8)
C10	0.0260 (12)	0.0163 (10)	0.0188 (11)	−0.0006 (9)	0.0007 (9)	−0.0027 (8)
C11	0.0203 (12)	0.0266 (12)	0.0309 (13)	−0.0041 (9)	0.0071 (9)	−0.0100 (10)
C12	0.0297 (14)	0.0410 (15)	0.0305 (14)	−0.0048 (11)	0.0132 (11)	−0.0093 (11)
C13	0.0227 (13)	0.0421 (16)	0.0498 (17)	−0.0012 (11)	0.0121 (11)	−0.0172 (13)
C14	0.0269 (12)	0.0171 (10)	0.0213 (11)	0.0005 (9)	0.0003 (9)	−0.0002 (9)
C15	0.0400 (14)	0.0167 (11)	0.0274 (13)	0.0031 (10)	0.0044 (10)	0.0016 (9)
C16	0.0257 (12)	0.0247 (12)	0.0309 (13)	0.0036 (10)	0.0010 (10)	0.0021 (10)
C17	0.0279 (12)	0.0158 (11)	0.0206 (11)	−0.0004 (9)	0.0023 (9)	0.0028 (8)
C18	0.0282 (13)	0.0198 (11)	0.0290 (13)	−0.0021 (9)	0.0081 (10)	0.0020 (9)
C19	0.0439 (15)	0.0271 (13)	0.0193 (12)	0.0006 (11)	0.0042 (10)	0.0052 (9)
O1	0.0551 (13)	0.0261 (10)	0.0424 (11)	−0.0098 (9)	−0.0069 (9)	0.0031 (9)
O2	0.0513 (14)	0.0450 (12)	0.0393 (12)	−0.0076 (10)	0.0015 (10)	−0.0159 (10)

### Geometric parameters (Å, º)

**Table:**

Cu1—N4^i^	1.9781 (18)	C9—H9B	0.9700
Cu1—N1	1.9929 (17)	C10—H10A	0.9700
Cu1—N5	2.0686 (17)	C10—H10B	0.9700
Cu1—N7	2.0740 (17)	C11—C13	1.527 (3)
Cu1—N6	2.2153 (18)	C11—C12	1.527 (3)
Ni1—C3	1.861 (2)	C11—H11	0.9800
Ni1—C4	1.864 (2)	C12—H12A	0.9600
Ni1—C2	1.866 (2)	C12—H12B	0.9600
Ni1—C1	1.871 (2)	C12—H12C	0.9600
N1—C1	1.148 (3)	C13—H13A	0.9600
N2—C2	1.144 (3)	C13—H13B	0.9600
N3—C3	1.147 (3)	C13—H13C	0.9600
N4—C4	1.147 (3)	C14—C16	1.520 (3)
N4—Cu1^i^	1.9781 (18)	C14—C15	1.528 (3)
N5—C10	1.488 (2)	C14—H14	0.9800
N5—C5	1.506 (2)	C15—H15A	0.9600
N5—C17	1.517 (3)	C15—H15B	0.9600
N6—C6	1.482 (3)	C15—H15C	0.9600
N6—C7	1.485 (3)	C16—H16A	0.9600
N6—C11	1.507 (3)	C16—H16B	0.9600
N7—C8	1.493 (3)	C16—H16C	0.9600
N7—C9	1.496 (2)	C17—C19	1.520 (3)
N7—C14	1.517 (3)	C17—C18	1.527 (3)
C5—C6	1.517 (3)	C17—H17	0.9800
C5—H5A	0.9700	C18—H18A	0.9600
C5—H5B	0.9700	C18—H18B	0.9600
C6—H6A	0.9700	C18—H18C	0.9600
C6—H6B	0.9700	C19—H19A	0.9600
C7—C8	1.526 (3)	C19—H19B	0.9600
C7—H7A	0.9700	C19—H19C	0.9600
C7—H7B	0.9700	O1—H1A	0.851 (9)
C8—H8A	0.9700	O1—H1B	0.846 (9)
C8—H8B	0.9700	O2—H2A	0.856 (10)
C9—C10	1.509 (3)	O2—H2B	0.861 (10)
C9—H9A	0.9700		
			
N4^i^—Cu1—N1	87.71 (7)	C10—C9—H9A	109.4
N4^i^—Cu1—N5	161.08 (7)	N7—C9—H9B	109.4
N1—Cu1—N5	94.59 (6)	C10—C9—H9B	109.4
N4^i^—Cu1—N7	93.53 (6)	H9A—C9—H9B	108.0
N1—Cu1—N7	177.96 (7)	N5—C10—C9	110.39 (17)
N5—Cu1—N7	84.76 (6)	N5—C10—H10A	109.6
N4^i^—Cu1—N6	111.86 (8)	C9—C10—H10A	109.6
N1—Cu1—N6	92.05 (7)	N5—C10—H10B	109.6
N5—Cu1—N6	86.86 (7)	C9—C10—H10B	109.6
N7—Cu1—N6	85.98 (6)	H10A—C10—H10B	108.1
C3—Ni1—C4	89.27 (9)	N6—C11—C13	113.30 (18)
C3—Ni1—C2	89.03 (9)	N6—C11—C12	111.79 (18)
C4—Ni1—C2	172.72 (9)	C13—C11—C12	110.86 (19)
C3—Ni1—C1	177.13 (9)	N6—C11—H11	106.8
C4—Ni1—C1	93.60 (8)	C13—C11—H11	106.8
C2—Ni1—C1	88.13 (9)	C12—C11—H11	106.8
C1—N1—Cu1	165.89 (17)	C11—C12—H12A	109.5
C4—N4—Cu1^i^	167.12 (16)	C11—C12—H12B	109.5
C10—N5—C5	109.71 (15)	H12A—C12—H12B	109.5
C10—N5—C17	110.33 (16)	C11—C12—H12C	109.5
C5—N5—C17	110.65 (15)	H12A—C12—H12C	109.5
C10—N5—Cu1	105.55 (11)	H12B—C12—H12C	109.5
C5—N5—Cu1	107.08 (13)	C11—C13—H13A	109.5
C17—N5—Cu1	113.32 (12)	C11—C13—H13B	109.5
C6—N6—C7	113.06 (16)	H13A—C13—H13B	109.5
C6—N6—C11	109.99 (16)	C11—C13—H13C	109.5
C7—N6—C11	113.98 (17)	H13A—C13—H13C	109.5
C6—N6—Cu1	98.69 (12)	H13B—C13—H13C	109.5
C7—N6—Cu1	104.33 (12)	N7—C14—C16	111.39 (17)
C11—N6—Cu1	115.81 (12)	N7—C14—C15	114.21 (17)
C8—N7—C9	111.17 (16)	C16—C14—C15	109.64 (18)
C8—N7—C14	110.08 (16)	N7—C14—H14	107.1
C9—N7—C14	111.30 (16)	C16—C14—H14	107.1
C8—N7—Cu1	101.33 (12)	C15—C14—H14	107.1
C9—N7—Cu1	109.08 (12)	C14—C15—H15A	109.5
C14—N7—Cu1	113.49 (12)	C14—C15—H15B	109.5
N1—C1—Ni1	173.96 (18)	H15A—C15—H15B	109.5
N2—C2—Ni1	175.5 (2)	C14—C15—H15C	109.5
N3—C3—Ni1	179.7 (2)	H15A—C15—H15C	109.5
N4—C4—Ni1	172.6 (2)	H15B—C15—H15C	109.5
N5—C5—C6	114.07 (17)	C14—C16—H16A	109.5
N5—C5—H5A	108.7	C14—C16—H16B	109.5
C6—C5—H5A	108.7	H16A—C16—H16B	109.5
N5—C5—H5B	108.7	C14—C16—H16C	109.5
C6—C5—H5B	108.7	H16A—C16—H16C	109.5
H5A—C5—H5B	107.6	H16B—C16—H16C	109.5
N6—C6—C5	114.12 (17)	N5—C17—C19	113.04 (17)
N6—C6—H6A	108.7	N5—C17—C18	111.13 (16)
C5—C6—H6A	108.7	C19—C17—C18	109.45 (18)
N6—C6—H6B	108.7	N5—C17—H17	107.7
C5—C6—H6B	108.7	C19—C17—H17	107.7
H6A—C6—H6B	107.6	C18—C17—H17	107.7
N6—C7—C8	112.05 (17)	C17—C18—H18A	109.5
N6—C7—H7A	109.2	C17—C18—H18B	109.5
C8—C7—H7A	109.2	H18A—C18—H18B	109.5
N6—C7—H7B	109.2	C17—C18—H18C	109.5
C8—C7—H7B	109.2	H18A—C18—H18C	109.5
H7A—C7—H7B	107.9	H18B—C18—H18C	109.5
N7—C8—C7	113.29 (17)	C17—C19—H19A	109.5
N7—C8—H8A	108.9	C17—C19—H19B	109.5
C7—C8—H8A	108.9	H19A—C19—H19B	109.5
N7—C8—H8B	108.9	C17—C19—H19C	109.5
C7—C8—H8B	108.9	H19A—C19—H19C	109.5
H8A—C8—H8B	107.7	H19B—C19—H19C	109.5
N7—C9—C10	111.25 (16)	H1A—O1—H1B	108.6 (15)
N7—C9—H9A	109.4	H2A—O2—H2B	107.6 (15)
			
N4^i^—Cu1—N1—C1	90.8 (7)	C4—Ni1—C3—N3	117 (40)
N5—Cu1—N1—C1	−108.0 (7)	C2—Ni1—C3—N3	−56 (40)
N7—Cu1—N1—C1	−37 (2)	C1—Ni1—C3—N3	−65 (40)
N6—Cu1—N1—C1	−21.0 (7)	Cu1^i^—N4—C4—Ni1	−54.8 (18)
N4^i^—Cu1—N5—C10	−60.4 (2)	C3—Ni1—C4—N4	−72.1 (13)
N1—Cu1—N5—C10	−156.75 (13)	C2—Ni1—C4—N4	4.4 (18)
N7—Cu1—N5—C10	25.19 (12)	C1—Ni1—C4—N4	108.0 (13)
N6—Cu1—N5—C10	111.44 (13)	C10—N5—C5—C6	−132.57 (18)
N4^i^—Cu1—N5—C5	−177.27 (18)	C17—N5—C5—C6	105.5 (2)
N1—Cu1—N5—C5	86.39 (12)	Cu1—N5—C5—C6	−18.5 (2)
N7—Cu1—N5—C5	−91.67 (12)	C7—N6—C6—C5	64.5 (2)
N6—Cu1—N5—C5	−5.42 (12)	C11—N6—C6—C5	−166.80 (17)
N4^i^—Cu1—N5—C17	60.4 (3)	Cu1—N6—C6—C5	−45.18 (18)
N1—Cu1—N5—C17	−35.91 (14)	N5—C5—C6—N6	47.3 (2)
N7—Cu1—N5—C17	146.03 (14)	C6—N6—C7—C8	−127.45 (19)
N6—Cu1—N5—C17	−127.72 (13)	C11—N6—C7—C8	106.0 (2)
N4^i^—Cu1—N6—C6	−155.83 (11)	Cu1—N6—C7—C8	−21.3 (2)
N1—Cu1—N6—C6	−67.47 (12)	C9—N7—C8—C7	65.3 (2)
N5—Cu1—N6—C6	27.02 (12)	C14—N7—C8—C7	−170.92 (17)
N7—Cu1—N6—C6	111.98 (12)	Cu1—N7—C8—C7	−50.51 (18)
N4^i^—Cu1—N6—C7	87.55 (13)	N6—C7—C8—N7	51.3 (2)
N1—Cu1—N6—C7	175.91 (13)	C8—N7—C9—C10	−133.78 (18)
N5—Cu1—N6—C7	−89.60 (13)	C14—N7—C9—C10	103.11 (19)
N7—Cu1—N6—C7	−4.64 (13)	Cu1—N7—C9—C10	−22.9 (2)
N4^i^—Cu1—N6—C11	−38.57 (16)	C5—N5—C10—C9	70.3 (2)
N1—Cu1—N6—C11	49.79 (15)	C17—N5—C10—C9	−167.58 (15)
N5—Cu1—N6—C11	144.28 (15)	Cu1—N5—C10—C9	−44.80 (18)
N7—Cu1—N6—C11	−130.76 (15)	N7—C9—C10—N5	46.1 (2)
N4^i^—Cu1—N7—C8	−83.12 (13)	C6—N6—C11—C13	−57.2 (2)
N1—Cu1—N7—C8	44.1 (19)	C7—N6—C11—C13	71.0 (2)
N5—Cu1—N7—C8	115.77 (13)	Cu1—N6—C11—C13	−167.98 (15)
N6—Cu1—N7—C8	28.57 (12)	C6—N6—C11—C12	176.65 (17)
N4^i^—Cu1—N7—C9	159.55 (13)	C7—N6—C11—C12	−55.2 (2)
N1—Cu1—N7—C9	−73.2 (19)	Cu1—N6—C11—C12	65.9 (2)
N5—Cu1—N7—C9	−1.55 (13)	C8—N7—C14—C16	169.88 (17)
N6—Cu1—N7—C9	−88.76 (14)	C9—N7—C14—C16	−66.4 (2)
N4^i^—Cu1—N7—C14	34.84 (14)	Cu1—N7—C14—C16	57.11 (19)
N1—Cu1—N7—C14	162.1 (19)	C8—N7—C14—C15	−65.2 (2)
N5—Cu1—N7—C14	−126.26 (14)	C9—N7—C14—C15	58.5 (2)
N6—Cu1—N7—C14	146.53 (14)	Cu1—N7—C14—C15	−178.02 (14)
Cu1—N1—C1—Ni1	41 (2)	C10—N5—C17—C19	−53.0 (2)
C3—Ni1—C1—N1	32 (3)	C5—N5—C17—C19	68.6 (2)
C4—Ni1—C1—N1	−149.3 (18)	Cu1—N5—C17—C19	−171.15 (14)
C2—Ni1—C1—N1	23.7 (18)	C10—N5—C17—C18	70.5 (2)
C3—Ni1—C2—N2	110 (3)	C5—N5—C17—C18	−167.91 (16)
C4—Ni1—C2—N2	33 (3)	Cu1—N5—C17—C18	−47.63 (19)
C1—Ni1—C2—N2	−71 (3)		

Symmetry code: (i) −*x*, −*y*+1, −*z*+1.

### Hydrogen-bond geometry (Å, º)

**Table:**

*D*—H···*A*	*D*—H	H···*A*	*D*···*A*	*D*—H···*A*
O1—H1*A*···N3^ii^	0.85 (2)	2.02 (2)	2.874 (3)	179 (3)
O1—H1*B*···O2^iii^	0.85 (2)	1.92 (2)	2.745 (3)	165 (2)
O2—H2*A*···N2	0.86 (2)	1.98 (2)	2.831 (3)	171 (3)
O2—H2*B*···O1^iv^	0.86 (3)	1.92 (3)	2.775 (3)	174 (2)

Symmetry codes: (ii) *x*, −*y*+1/2, *z*−1/2; (iii) −*x*+1, −*y*+1, −*z*+1; (iv) *x*, *y*, *z*+1.
